# Acute Cholecystitis, A Rare Complication Following Routine Colonoscopy: Case Series and Literature Review

**DOI:** 10.7759/cureus.10877

**Published:** 2020-10-10

**Authors:** Danial H Shaikh, Kishore Kumar, Harish Patel, Shehriyar Mehershanhi, Jasbir Makker

**Affiliations:** 1 Gastroenterology, BronxCare Health System, Bronx, USA

**Keywords:** colonoscopy complications, acute calculous cholecystitis, abdominal pain, gall bladder diseases and gallstones, gastroenterology and endoscopy

## Abstract

Colonoscopy is a commonly performed low-risk gastrointestinal procedure that may rarely result in a serious complication. Patients presenting with abdominal pain and fever after colonoscopy may have acute cholecystitis. The underlying mechanisms are unclear. Such patients usually present within 72 hours of the procedure. Treatment includes intravenous antibiotics and cholecystectomy. We present our experience of two such cases; a 56-year-old man and a 21-year-old man, both of whom developed acute calculus cholecystitis within 48 hours after a routine colonoscopy. Their symptoms resolved after cholecystectomy.

## Introduction

Colonoscopy is generally considered a safe and low-risk gastrointestinal procedure, and as such severe adverse events are rare. However, with approximately more than 15 million colonoscopies being performed each year in the United States (US) alone, events such as bleeding, perforation, and mortality do occur [1]. Mild abdominal pain/discomfort immediately after a colonoscopy is not rare, occurring anywhere between 2.5% to 11% of the cases [2]. Though it may have a host of etiologies, it is most commonly a result of air insufflation, endoscope looping, and/or manual pressure maneuvers used during a colonoscopy. Rarely, abdominal pain may be severe and unrelenting. In such cases, one should pursue abdominal imaging, in order to rule out colonic perforation. Severe pain after colonoscopy may very rarely occur due to acute cholecystitis, especially with concomitant fever and abnormal liver enzymes. With the increasing population of US adults older than 50 years of age, the number of annual screening colonoscopies has also risen (from 34% in 2000 to 63% in 2015) and is expected to increase further [3]. Therefore, knowledge of rare adverse events such as acute cholecystitis is paramount, as prompt recognition may alter management and outcomes. Only a handful of cases of acute cholecystitis as an adverse event of colonoscopy have been reported thus far. We present our experience with two such cases.

## Case presentation

Case 1

A 56-year-old man with cognitive delay was brought to the emergency department with abdominal pain for two days. The patient had undergone a colonoscopy for colorectal cancer screening two days prior to presentation. The quality of bowel preparation was suboptimal, however, the procedure was completed until the cecum without any technical difficulties. During the procedure, no manual pressure or change in patient position was required. No polyps were found and therefore no therapeutic interventions were performed. The patient described the pain as sharp, constant, non-radiating, and located in the epigastrium and right upper quadrant of the abdomen. He endorsed the onset of the pain on the eve following his procedure and denied any previous history of similar pain. He had no associated symptoms of fever, nausea, vomiting, constipation, and diarrhea. His other medical comorbidities included hypertension, diabetes mellitus, dyslipidemia, and hypothyroidism. He had undergone a left nephrectomy six years earlier for a stage one renal cell carcinoma. His family history was unremarkable, and he denied the use of any illicit drugs. He lived with his sister who corroborated his history. 

**Figure 1:**
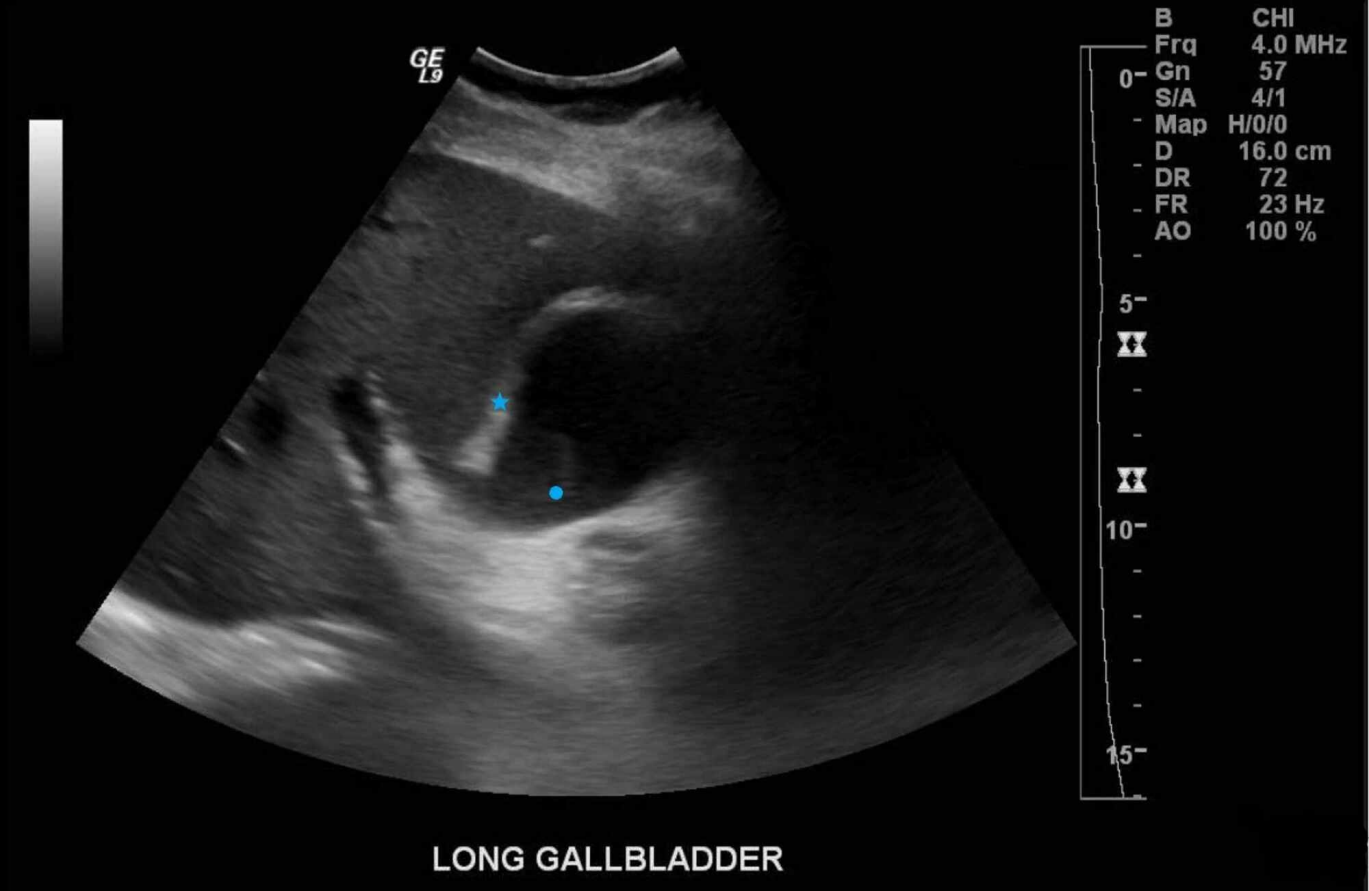
Ultrasound of the abdomen demonstrating gallbladder wall thickening (star) and layering sludge (circle), concerning for acute cholecystitis

**Figure 2:**
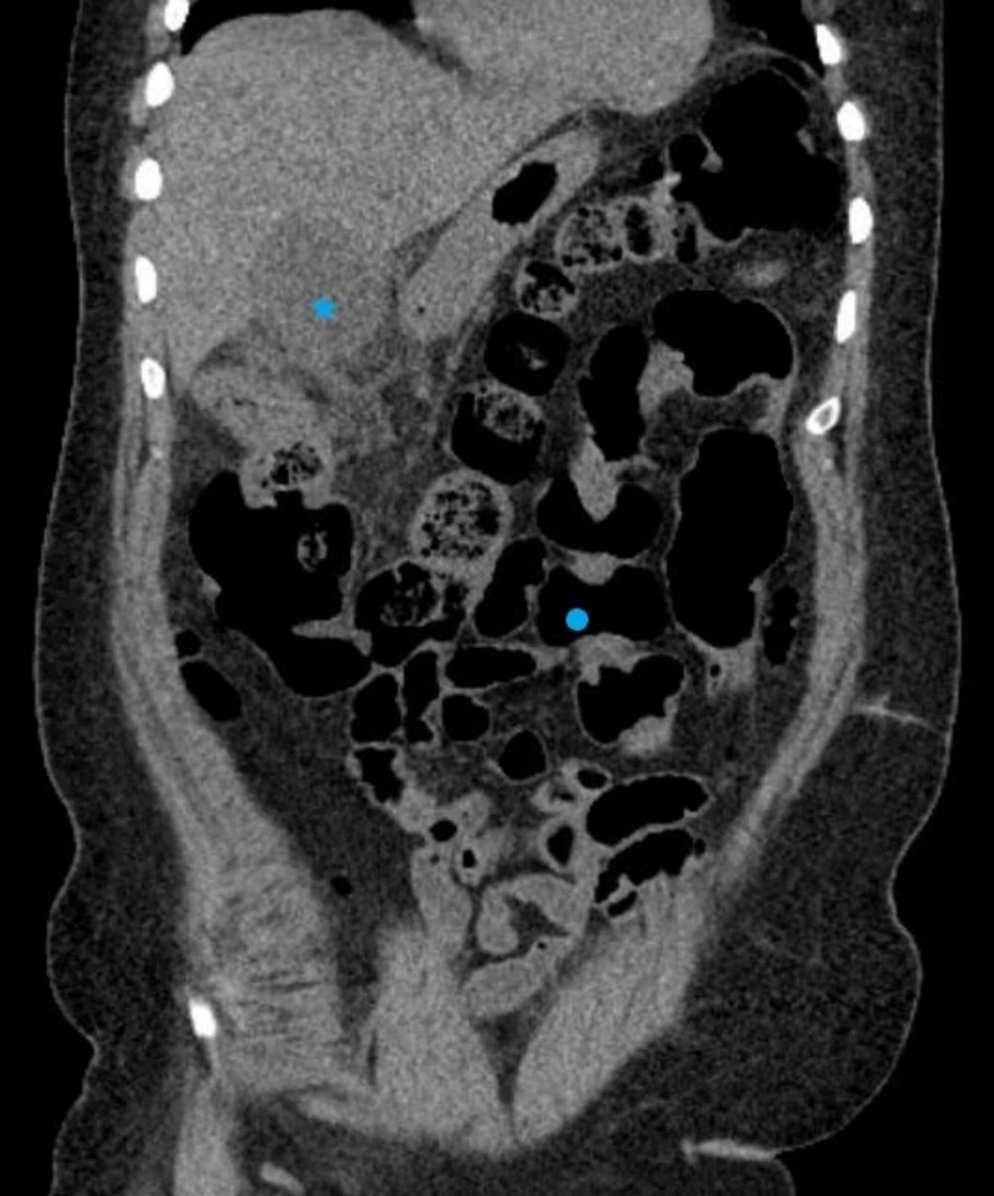
CT scan of the abdomen demonstrating a distended gallbladder (star) and no signs of large bowel perforation (circle)

On arrival to the emergency department, he was comfortable, in no apparent distress. Vital signs showed tachycardia with a heart rate of 115 beats per minute, a temperature of 99°F, blood pressure of 151/92 mmHg, and oxygen saturation of 96% on room air. His abdomen was non-distended with normal bowel sounds. There was mild tenderness in the right upper quadrant of the abdomen on palpation; no abdominal rigidity, guarding or organomegaly was appreciated. The neurologic examination was at baseline. Cardiopulmonary and skin examination were within normal limits. Initial laboratory workup revealed the presence of leukocytosis, elevated transaminases, and total bilirubin (Table 1). He underwent an ultrasound of the abdomen which demonstrated layering of gall bladder sludge, presence of pericholecystic fluid, a distended and thickened wall of the gall bladder measuring seven millimeters, and a common bile duct measuring five millimeters in diameter (Figure 1). Sonographic Murphy’s sign was positive. A subsequent computed tomography (CT) scan of the abdomen did not reveal any large bowel abnormalities (Figure 2). The patient was started on broad-spectrum antibiotics, intravenous fluids, and kept nil per orally. An emergent laparoscopic cholecystectomy was performed. Intraoperatively, a thickened and pus-filled gallbladder with an intact gallbladder wall was removed. The patient had uneventful postoperative recovery with normalization of liver function tests and resolution of leukocytosis.

**Table 1:** Case 1 admission laboratory values g = grams, mg = miligrams, ul = microliters, k = thousand, dl = deciliter, L = Liters, WBC = White Blood Cell Count, BUN = Blood Urea Nitrogen

Hemoglobin (g/dl)	14.2	Aalanine aminotransferase (unit/L)	150
Hematocrit (%)	45.9	Aspartate tranaminases (unit/L)	121
Platelet count (k/ul)	205	Total Bilirubin (mg/dl)	4.0
WBC (k/ul)	17.6	Direct Bilirubin (mg/dl)	2.5
BUN (mg/dl)	13	Alkaline phosphatase (unit/L)	125
Creatinine (mg/dl)	1.3	Lipase (unit/L)	36
Blood culture	No growth	Urine culture	No growth

Case 2

A 21-year-old man with dyspepsia, gastroesophageal reflux disease, and anxiety, presented to the emergency department with a complaint of right upper quadrant abdominal pain for the past eight hours. As per the patient, the pain started gradually after having dinner and increased in intensity until it was unbearable, prompting him to seek medical attention. He described the pain as sharp and constant in nature that radiated to the back. It was associated with a feeling of nausea, however, he denied any vomiting. He denied any fever, diarrhea, constipation, or any signs of gastrointestinal blood loss. The patient was recently discharged from the same hospital a day earlier, where he was admitted for epigastric abdominal pain and fifty pounds of unintentional weight loss, over a period of four months. The epigastric pain was chronically present for the past six months. Prior testing had included a normal upper endoscopy.

**Figure 3:**
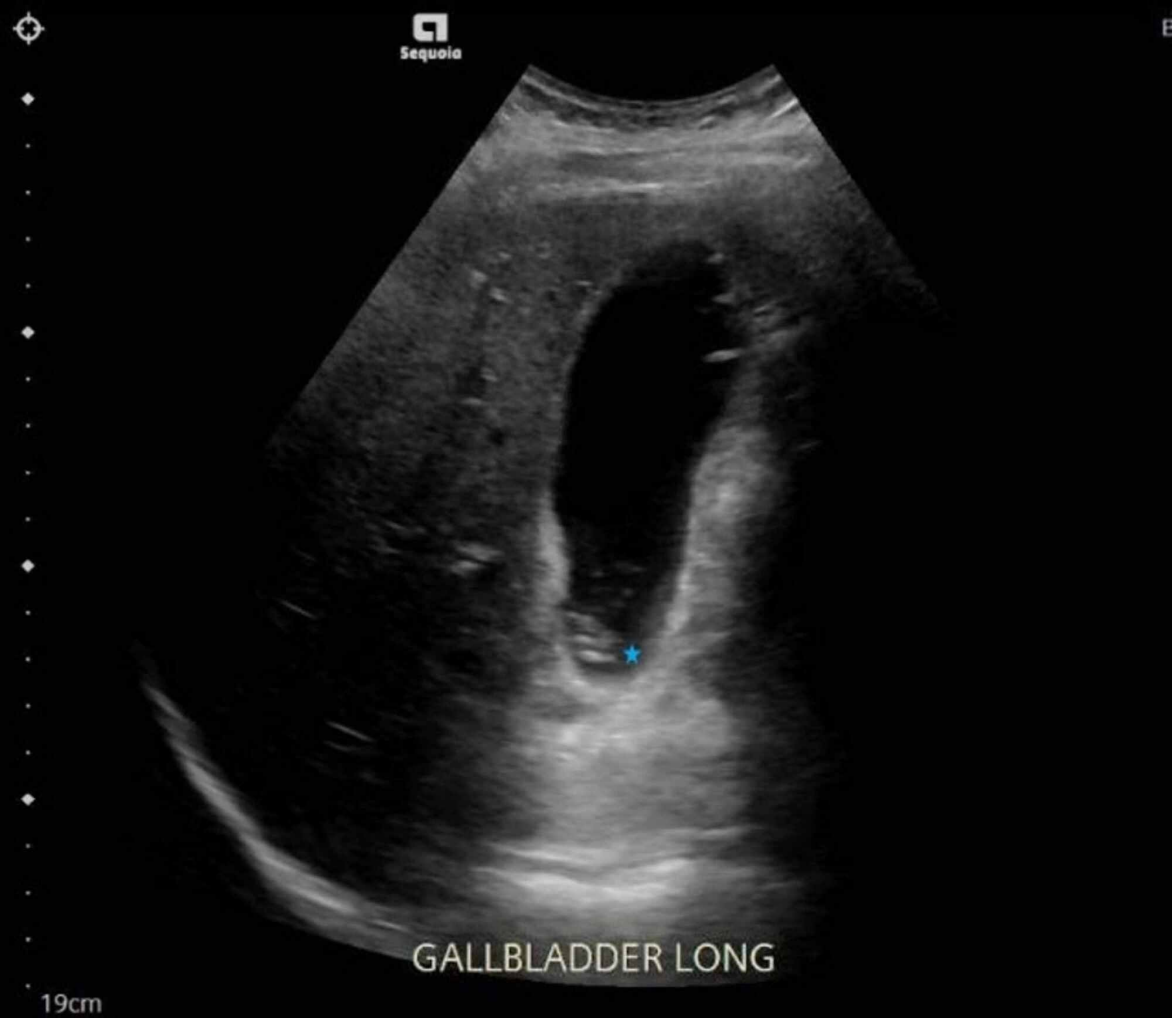
Ultrasound of the abdomen demonstrating gallstones (star) without evidence of acute cholecystitis

**Figure 4:**
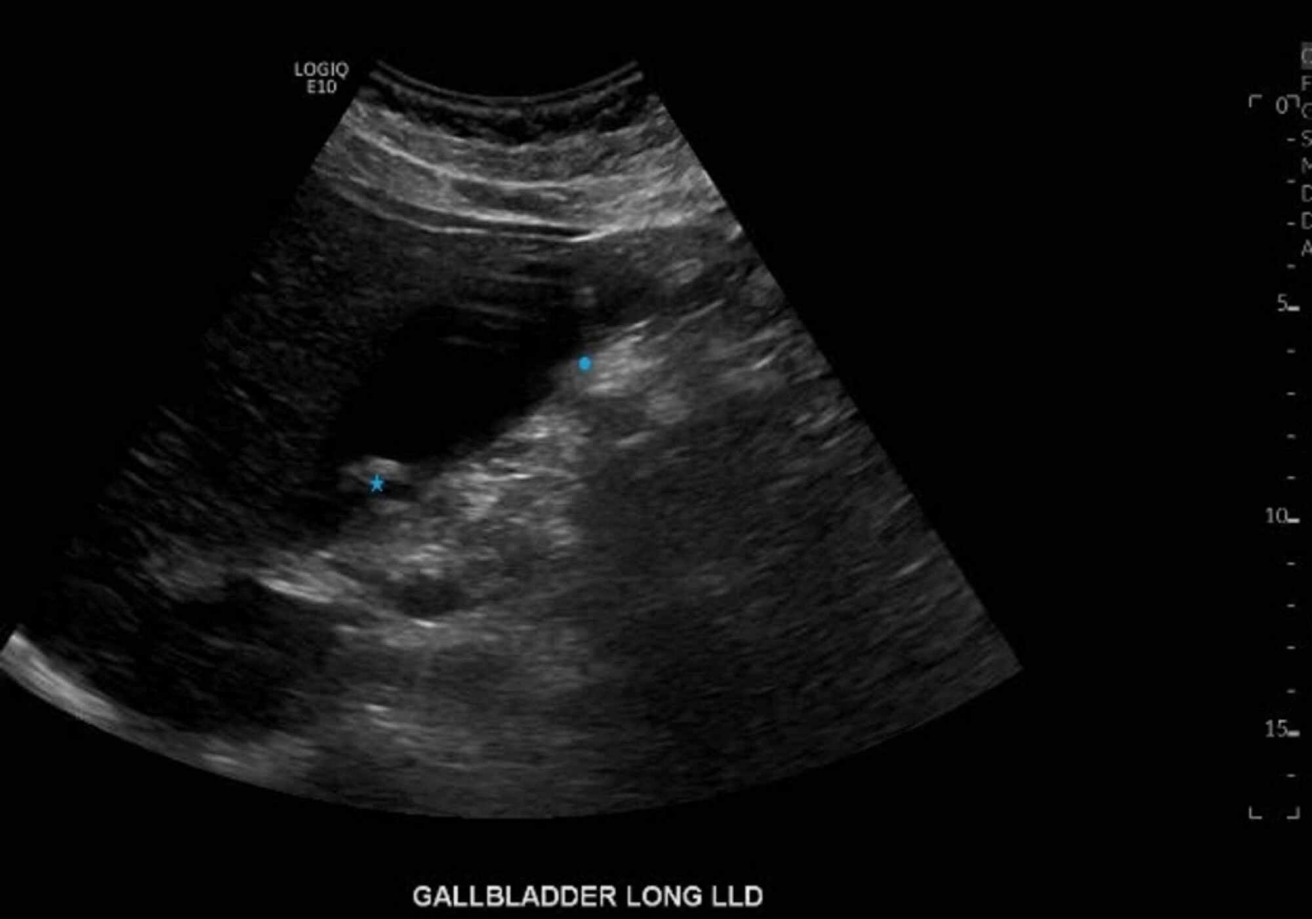
Ultrasound of the abdomen demonstrating multiple stones in the gallbladder (star) with mild prominence of the gallbladder wall (circle) concerning for acute cholecystitis

**Figure 5:**
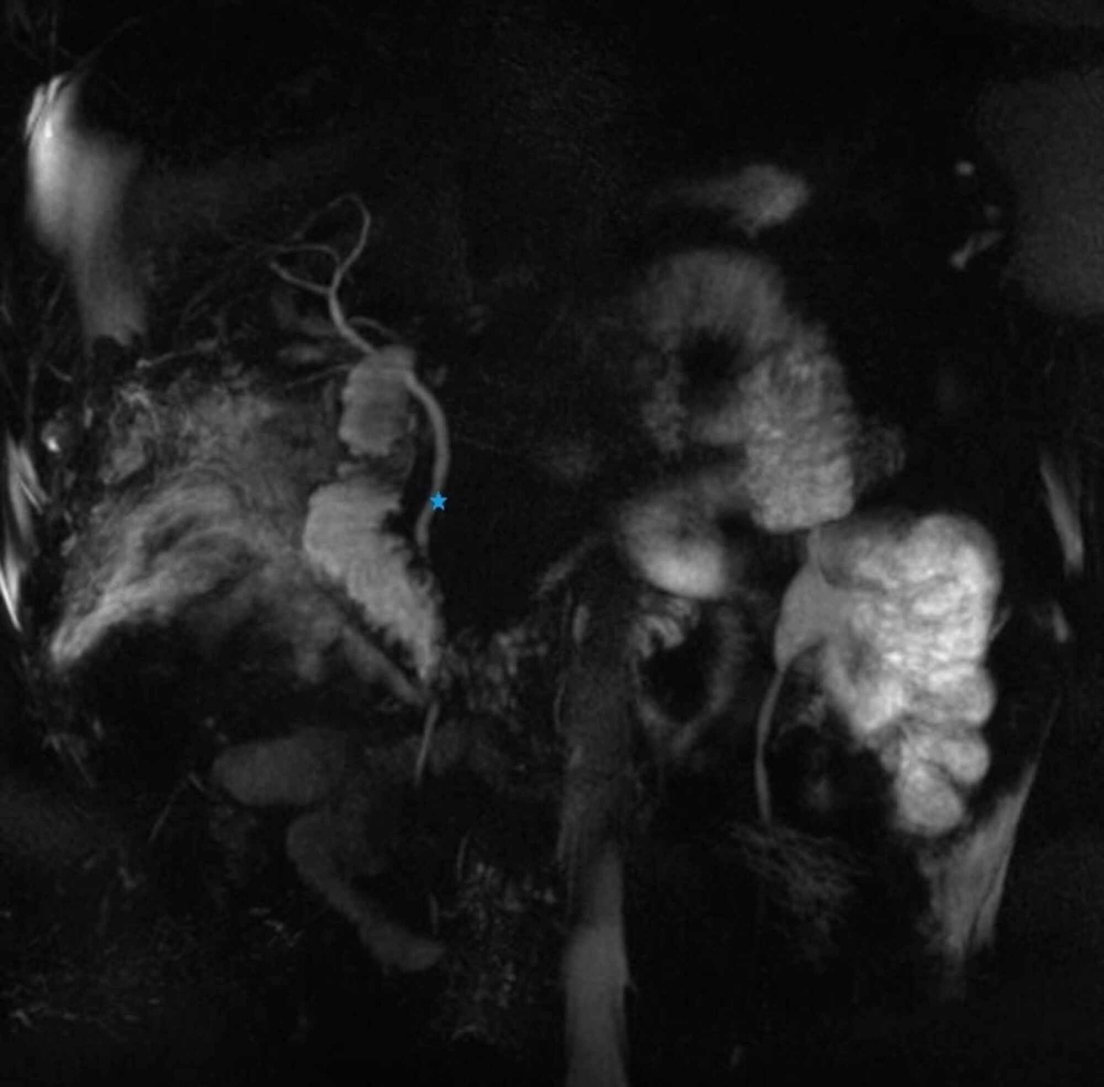
Magnetic resonance cholangiopancreatography demonstrating a normal common bile duct (star) without any filling defects

An ultrasound of the abdomen during that visit had demonstrated cholelithiasis without radiographic evidence of cholecystitis or biliary obstruction, and the common bile duct had measured three millimeters in diameter (Figure 3). For evaluation of weight loss, a push enteroscopy was performed, followed by a colonoscopy the next day. The push enteroscopy revealed patchy erythematous pre-pyloric mucosa, reported as foveolar hyperplasia on pathology. The colonoscopy was uneventful, the quality of the bowel preparation was good and the terminal ileum was intubated. No manual pressure or change in patient position was required. The patient was discharged home the next day after his colonoscopy, and upon discharge, he did not complain of any abdominal pain. He did not have any prior surgeries, did not smoke cigarettes, drink alcohol, or does drugs. His family history was largely non-significant and he did not travel anywhere recently. Vital signs during this second visit showed a heart rate of 82 bpm, a blood pressure of 140/69 mmHg, oxygen saturation of 96% on room air, and a temperature of 98.1°F. On physical examination, the patient was moaning in pain. The cardiopulmonary exam was unremarkable; however, his abdomen was tender to palpation in the right upper quadrant. Bowel sounds were normoactive and no guarding, rigidity, or organomegaly was elicited. No skin rashes were appreciated, and the neurological exam was within normal limits. An ultrasound of the abdomen this visit demonstrated multiple stones in the gallbladder with mild prominence of the gallbladder wall and a positive sonographic Murphy’s sign (Figure 4). The common bile duct was prominent measuring seven millimeters in diameter. A follow-up magnetic resonance cholangiopancreatography did not reveal a common bile duct stone (Figure 5). Initial laboratory investigations were unremarkable, except for a mildly elevated alanine aminotransferase level (Table 2). Upon admission, he was initially started on intravenous antibiotics and underwent a laparoscopic cholecystectomy the next day. Post-procedure his pain improved and he was eventually discharged home. Pathological studies of the surgical specimen were reported as acute on chronic cholecystitis and cholelithiasis.

**Table 2:** Case 2 admission laboratory values g = grams, mg = miligrams, ul = microliters, k = thousand, dl = deciliter, L = Liters, WBC = White Blood Cell Count, BUN = Blood Urea Nitrogen

Hemoglobin (g/dl)	14	Aalanine aminotransferase (unit/L)	46
Hematocrit (%)	40.2	Aspartate tranaminases (unit/L)	24
Platelet count (k/ul)	241	Total Bilirubin (mg/dl)	0.5
WBC (k/ul)	7.3	Direct Bilirubin (mg/dl)	0.2
BUN (mg/dl)	6	Alkaline phosphatase (unit/L)	71
Creatinine (mg/dl)	0.7	Lipase (unit/L)	26

## Discussion

Serious adverse events after a colonoscopy, described as bleeding, perforation, post-polypectomy syndrome, and cardiopulmonary adverse events related to moderate or deep sedation, have been estimated to be around 2.8 per thousand colonoscopies performed [4]. Other uncommon but severe complications attributed to colonoscopy include infection, splenic injury, colitis, gas explosion, and rarely death. Cholecystitis after colonoscopy is an extremely rare event with no estimates of prevalence available in current literature.

Post colonoscopy cholecystitis was first reported in 2001 by Milman and Goldenberg in a series of two patients [5]. After this initial report, an additional seven authors reported cholecystitis after colonoscopy [6-12] in a total of twelve patients (Table 3). The precise mechanism leading to cholecystitis after colonoscopy remains unknown. Several theories have been postulated, the most established of which suggests that bowel preparation prior to colonoscopy leads to dehydration, which in turn makes the bile lithogenic. Lithogenicity of the bile leads to stasis and obstruction of the cystic duct resulting in cholecystitis [5]. Another accepted theory takes into account the presence of pre-existing cholelithiasis, which due to the manipulation of internal viscera during colonoscopy, can become impacted in the gallbladder neck [9]. Yet another theory argues that manipulation of the patient during the procedure, such as applying abdominal pressure to accomplish advancement of the scope in technically difficult cases, or extensive manipulation of the scope within the colon, such as with looping, can cause bacterial translocation leading to cholecystitis [11]. A fourth, and less widely accepted theory, highlights the role of thermal injury and inflammation occurring after the use of electrosurgical current for polypectomy performed during a colonoscopy.

**Table 3:** Reported cases of cholecystitis after colonoscopy

Authors	Age	Gender	Polyp location	Intervention	Symptom onset after colonoscopy	Presenting complaint	Gallstone on imaging
Milman et al. 2001 [5]	58	Female	Not reported	Cold biopsy	<24 hours	Epigastric pain and fever	Yes
Milman et al. 2001 [5]	49	Female	None	Cold biopsy (random biopsy)	<24 hours	Epigastric pain	Yes
Fernandez-Martiınez et al. 2002 [11]	76	Male	Not reported	3 small polyps, cold biopsy	<24 hours	Right upper quadrant abdominal pain and fever	No
Aziz et al. 2007 [10]	63	Female	Not reported	Small polyps removed but intervention not reported	24 hours	Epigastric pain	Yes
Aziz et al. 2007 [10]	60	Male	Not reported	Polypectomy	72 hours	Epigastric pain	Yes
Maddur et al. 2011 [9]	70	Male	Not reported	Cold snare polypectomy and cold forceps	48 hours	Diffuse abdominal pain and fever followed by right upper quadrant pain	Yes
Maddur et al. 2011 [9]	70	Male	Descending colon	Cold snare and hot snare	72 hours	Epigastric pain	Yes
Maddur et al. 2011 [9]	57	Female	Ascending colon	Hot snare polypectomy	48 hours	Right sided abdominal pain and fever	Yes
Park et al. 2013 [8]	35	Male	Sigmoid	Hot snare polypectomy	48 hours	Epigastric pain and fever	Yes
Warfe et al. 2013 [7]	69	Female	None	None	<12 hours	Epigastric pain	No
Gorgan et al. 2016 [6]	57	Male	Rectosigmoid	Small polyp removed but intervention not reported	<24 hours	Right sided abdominal pain	Yes
Campbell et al. 2020 [12]	72	Male	Not Reported	None	<12 hours	Right Upper quadrant abdominal pain	No

We performed a detailed review of all the published cases in the literature, as summarized in Table 3. Cholecystitis typically occurred within 72 hours after colonoscopy. Most cases reported the presence of gallstones on imaging studies. On review, polypectomy seemed an unlikely contributor to cholecystitis as most reported cases had no interventions, and those that did were unable to find any intraoperative abnormalities of the colon adjacent to the polypectomy site [9]. The case by Warfe et al. support the theory of manipulation of adjacent abdominal organs as the underlying cause of cholecystitis during a colonoscopy. They describe torsion of a wandering gallbladder brought about by excessive manipulation in trying to overcome a difficult and tortuous colon [7]. Though the details of their colonoscopy are not mentioned, Fernandez-Martinez et al. [11] and Campbell et al. [12] reported growth of pathogenic gut flora outside the colon immediately after colonoscopy, presumably occurring via bacterial translocation. Campbell et al. [12] report a case of emphysematous cholecystitis in their patient secondary to extended-spectrum beta-lactamase-producing Escherichia coli, whereas, Fernandez-Martinez et al. [11] found evidence of Clostridium spp. and Enterococcus facecalis in the pericholecystic fluid of their patient with gangrenous cholecystitis. In all remaining cases [5,6,8-10], the most likely underlying pathophysiology is due to increased lithogenicity of bile, as a direct result of the fasting state and commonly used large volume laxatives consumed for bowel cleansing.

Though the exact mechanisms giving rise to acute cholecystitis after a colonoscopy remain unclear, it is worthy to note that both our patients had a similar presentation and clinical course to those cases described in the available literature. This perhaps suggests that there is a pattern to this rare adverse event and possibly refutes its presence as just mere coincidence. Both our patients presented within the 72-hour time frame and both had documented cholelithiasis on imaging, favoring theories of increased lithogenicity and/or stone impaction from scope manipulation, as the cause of cholecystitis.

It is important to consider post colonoscopy cholecystitis in patients who present with abdominal pain, fever, and elevated transaminases after the procedure. Since colonic perforation is more commonly observed after colonoscopy; it is important to exclude this possibility by obtaining imaging studies such as an X-ray or a CT scan of the abdomen. Once perforation is excluded, an ultrasound of the abdomen should be considered to evaluate for acute cholecystitis, particularly in patients with abnormal liver function tests. Initial treatment includes intravenous antibiotics, intravenous fluids, and nothing per oral. Cholecystectomy remains the mainstay of the treatment.

## Conclusions

Colonoscopy, a commonly performed gastrointestinal procedure, may rarely result in serious complications. Patients who present with abdominal pain and fever after colonoscopy may have acute cholecystitis, especially if the liver function tests are also abnormal. These patients usually present within 72 hours of the procedure. Several theories exist regarding the underlying mechanism, however, the exact cause remains unclear. Bowel perforation should be ruled out immediately after which an ultrasound of the abdomen should be pursued. Treatment includes intravenous antibiotics and cholecystectomy.
